# A Complicated Microbiology Diagnosis and Treatment of Tonsillopharyngitis in Pregnancy: A Case Report

**DOI:** 10.1155/carm/5675549

**Published:** 2025-09-22

**Authors:** Vasil Boyanov, Ivo Sirakov, Bilyana Sirakova, Liliya Boyanova, Raina Gergova

**Affiliations:** ^1^Department of Medical Microbiology, Medical University of Sofia, Sofia, Bulgaria; ^2^“AIPPMPDM”, Ltd., Sandanski 2800, Bulgaria

**Keywords:** pregnancy, sequencing, *Streptococcus anginosus*, tonsillopharyngitis

## Abstract

Pregnancy is a special period characterized by changes in the immune system as well as alteration of the microbiota predisposing to opportunistic infections, including tonsillopharyngitis. *Streptococcus anginosus* has numerous virulence factors, and it is associated with a variety of diseases. Being a part of the normal oral microbiota, it can be overlooked as a causative agent of infections in the oral cavity. A clinical case of a 29-year-old pregnant female who was allergic to penicillins and presented with recurrent tonsillopharyngitis has been described. Previous microbiological examinations have not shown any etiologic cause of patient's symptoms. Two species were isolated. *S. anginosus* and *S. oralis* were identified using various methods, including nucleotide sequencing. *S. anginosus* was defined as the causative agent. Both were susceptible to all tested antibiotics. *S. anginosus* was positive for the duplicated *sagA* gene, encoding streptolysin S, an important factor of virulence. Based on clinical symptoms, paraclinical tests, immune competence, and characteristics of the strain, a multifactorial evaluation is required to determine whether an opportunist is a causative agent. Moreover, treatment of pregnant patients, particularly with antibiotics, is limited, which leads to therapeutic difficulties.

## 1. Introduction

One of the most prevalent upper respiratory conditions is tonsillopharyngitis, which is an infection affecting both the pharyngeal wall and the tonsils. The infection can be categorized as either acute, subacute, or chronic, based on the duration time, specific causative agent, and the competence of the patient's immune system. Although some viruses and *Streptococcus pyogenes* are the most common pathogens implicated in the etiology of this disease, other streptococci may also be involved, especially in older children and adults [1, 2].

The *Streptococcus anginosus* group (SAG) consist of *Streptococcus anginosus*, *Streptococcus constellatus*, and *Streptococcus intermedius* [3]. Species of SAG are Gram-positive cocci with tendency to arrange in chains, nonmotile, facultative anaerobes. They form small colonies up to 0.5 mm with caramel-like odor of diacetyl and exhibits variable hemolysis (either alpha, beta, or gamma) [4, 5]. As biochemical properties, *S. anginosus* group bacteria are negative in catalase, Christie–Atkins–Munch–Petersen (CAMP), and pyrrolidonyl arylamidase (PYR) tests, as well as sorbitol and mannitol fermentation, while positive for Voges–Proskauer, arginine dihydrolase, and esculin hydrolysis [6]. According to Lancefield classification, SAG can be referred to as either group A, C, G, F, or N since it reacts with the respective antisera [7]. However, in some cases they do not carry any Lancefield group antigen [8].

SAG is an opportunistic pathogen, part of the normal microbiota of the oral cavity and upper respiratory tract, as well as the urogenital and gastrointestinal systems [9]. Streptococci are among the first microorganisms that the colonize the oral cavity of the neonate. Their strong biofilm-forming activity shapes the mouth and throat microbiota of the individual [10]. SAG is associated with pyogenic infections including abscesses with various localizations such as skin and soft tissue, the abdominal cavity, and the skeletal system [5]. It can cause tonsillitis and/or pharyngitis in adults [11]. As a complication, peritonsillar abscess may develop that is usually a mixed infection of SAG with anaerobic bacteria [6]. SAG is associated not only with infections but also with neoplastic diseases. Its relationship with oral and pharyngeal cancers was studied, and recently its association with gastric cancer was reported [12, 13].

*Streptococcus anginosus* shares some common virulence factors with *Streptococcus pyogenes* such as streptolysin, fibrinolysin, and M-protein. Even it can trigger nonsuppurative complications like acute glomerulonephritis [14]. The main factors of virulence are hemolysins, adhesins, DNases, toxins, pili, hyaluronidases, enolases, and superantigens. Isolates that cause complete β-hemolysis are considered potentially pathogenic. This characteristic is determined by the production of streptolysin S (encoded by the *sagA* gene), similar to *S. pyogenes* [3, 15, 16].

Pregnancy represents a distinctive period in a woman's life characterized by numerous changes in their body. During that period the immune system of the mother shifts towards Th2 exhibiting tolerance towards the allogenic fetus. The function of neutrophils and monocytes is altered, and there is data that immunoglobulin levels decrease [17]. During pregnancy the oral microbiome increases its load including the number of streptococci [18]. Expectant mothers have limitations in their treatment since some medications are known to cause adverse events, while the safety of others has not been tested in pregnancy. Penicillins and cephalosporins are the primary options as etiological treatment in bacterial infections in pregnant women [19]. Patients with severe Type I allergies to penicillins should not be administered cephalosporins either. In such cases macrolides, lincosamides, or vancomycin are relevant [20].

## 2. The Case Report

### 2.1. Case Presentation

**Figure 1:**
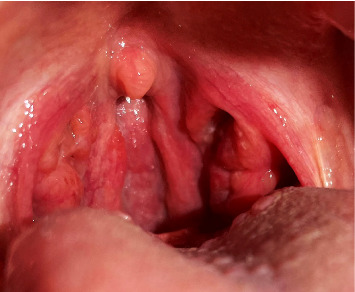
Inflammation of pharyngeal mucosa and tonsils during first trimester.

**Figure 2:**
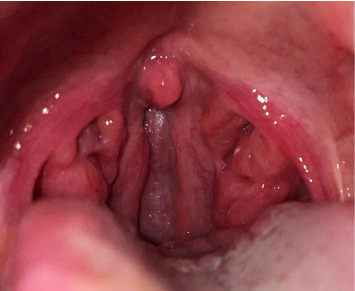
Tonsillolith.

We present a case of a 29-year-old pregnant female with no comorbidity. She self-reported penicillin allergy that had manifested with coughing, dyspnea, itchy rash, and red itchy eyes. During the first trimester (eighth gestation week), she had a common cold that resolved after two, 3 days period without treatment. Subsequently, 2 days later, she started to experience pain when swallowing and malaise. The examination revealed red pharyngeal mucosa with prominent lymph follicles; the tonsils were red and swollen (Figure 1). There were no palpable enlarged lymph nodes. The patient was subfebrile. Symptoms went away 10 days later without antibiotic treatment. The condition recurred 2 more times during pregnancy. Meanwhile, painless tonsilloliths (tonsil stones) were regularly observed (Figure 2).

She underwent the following tests: complete blood count (normal, no left shift), erythrocytes sedimentation rate (40 mm/h), and CRP (13 mg/L). Immunological tests were done for detection of antibodies against Epstein–Barr virus (IgM, IgG, and EBNA) and Cytomegalovirus (IgM and IgG). They showed no evidence of a current or a recent infection. Three weeks after the initial symptoms, the antistreptolysin O titer was tested with a negative result. The condition recurred two more times until the end of the pregnancy with slightly elevated inflammation-related paraclinical results.

### 2.2. Etiological Diagnosis

Microbiological throat swab was analyzed along with the patient's physical examination during the course of the disease. Two bacterial species were predominant, while there was no growth of *Candida* spp. On blood agar the colonies had the typical morphology of beta and alpha hemolytic streptococci, respectively. The microbial count performed by modifying a method described by Reynolds [21] presented overgrowth of very small β-hemolytic colonies with over 10^5^ cfu/mL and α-hemolytic ones with 10^4^ cfu/mL. The conventional tests (PYR, CAMP, and bacitracin susceptibility) pointed to the beta-hemolytic *Streptococcus* being a non-A-non-B species. The latex-agglutination test for Lancefield serological group (PathoDxtra Strep Grouping Kit, Oxoid, Thermo Fisher Scientific, Inc., USA) showed agglutination in the beta-hemolytic strain (group F), but not in the alpha-hemolytic isolate. We performed BD PhoenixTM M50 (Becton, Dickinson and Company, USA) biochemical identification. The results for the two species were SAG and the *S. mitis* group, with confidence levels of 93% and 95%, respectively.

The species were further detailed through sequencing. Protocols from the PCR amplification and nucleotide sequencing reaction steps were previously described by Lane and Sirakov [22, 23]. The obtained sequences were processed using MEGA-X software [24] and analyzed for close homology using the Basic Local Alignment Search Tool (BLAST) available at the National Center for Biotechnology Information (NCBI) (http://www.ncbi.nlm.nih.gov/BLAST). The processed sequences (derived from α- and β-hemolytic isolates) demonstrated a 100% identity to the *S. anginosus* and *S. oralis* sequences found in the GenBank database, NCBI.

According to previously published PCR primers and conditions [15], *S. anginosus* tested positive for the *sagA* gene. Given that the screening primers utilized in the referenced study [15] were exclusively for the identification of SAG genotype II, characterized as the *sag* gene cluster containing duplicated *sagA* copies, and since we yielded positive PCR results in our research, we concluded that the duplicated *sagA* gene is present in the current isolate.

Antimicrobial susceptibility testing was performed using the Kirby–Bauer disk diffusion method. For interpretations of the results of antibiotic testing, EUCAST recommendations were used [25]. Both isolated microorganisms showed susceptibility to all tested antibiotics, including beta-lactams, lincosamides, and vancomycin.

To demonstrate the etiological value of the suspected pathogen, a passive hemagglutination serological test was performed by modifying a method described by Gelil [26]. With the reaction, a significant rise in the titer was registered within 3 weeks. Through this reaction, a titer increase of 16 (normal < 2) was observed against *S. anginosus*, although a reduction in its microbial count was observed. Meanwhile, no elevated titer was noted for *S. oralis*, which maintained a score of 2. This finding confirms the emergence of an infection caused by *S. anginosus*, but it does not indicate an immune response against *S. oralis*, thereby excluding its role as an etiological agent.

### 2.3. Treatment and Outcome

The attending physician prescribed nonantibiotic therapy due to the subacute symptoms, relatively preserved general condition, paraclinical findings, anamnesis of allergic reactions, and the patient's reluctancy to take antibiotics during pregnancy. Patient was treated with a lactoferrin supplement indicated for boosting the immunity in pregnancy. Oral probiotic was taken and gargles were done.

Although the condition recurred throughout the pregnancy, there were no complications related to pregnancy and delivery.

## 3. Discussion

Tonsillopharyngitis can be caused by either bacteria or viruses such as Epstein–Barr and Cytomegalovirus [27]. The exclusion of the latter was essential since it is a TORCH agent [28]. However, repetitive tonsillitis is predominantly bacterial [29]. Probably the altered oral microbiota became the main reason for the persistent secondary bacterial infection. According to some authors, changes in oral microbiota are most prominent in the first trimester [30].

In some laboratories non-A-non-B streptococci are reported as normal microbiota. The primary reason is that, according to some authors, only *S. pyogenes* requires an etiological diagnosis and specific treatment. In this way, other streptococci that are resistant to bacitracin can be overlooked as causative agents of oral infections [31, 32].

Beta-hemolytic *S. anginosus* carries streptolysin S encoding gene cluster, a reason for cytotoxicity on human cells in vitro. So, presumptively, they are considered more virulent than alpha and gamma hemolytic strains from the species [3, 15]. In our study, the *S. anginosus* strain tested positive for the duplicated *sagA* gene, which determines a higher pathogenic potential, showing that this microorganism is likely the causative agent of the infection.

According to some authors, antimicrobial treatment has not been shown to be beneficial in acute tonsillitis or pharyngitis caused by streptococci other than GAS [33]. Some antibiotics, including penicillins, at standard doses reach insufficient concentrations in tonsillar surface fluid as well as in the tonsil tissues [1, 34].

Recommended medications to treat respiratory infections during pregnancy include penicillins, macrolides, and clindamycin [19, 20]. The EUCAST [25] indicates that there is insufficient evidence that macrolides can be used to treat SAG-associated infections. Another option is clindamycin, which is generally considered safe and effective in pregnancy. Since clindamycin inhibits the production of bacterial toxins, it is also preferred in toxin-mediated infections [35, 36]. However, it is classified as a nitrosatable amide and is associated with teratogenic effects such as isolated cleft palate, cardiac, and musculoskeletal malformations [19, 37, 38]. Thereafter, selecting the appropriate antibiotic treatment for pregnant patients with a history of allergic reactions to penicillins poses a significant challenge.

Alpha-hemolytic *Streptococcus oralis*, part of the *S. mitis* group, is one of the most prevalent commensal bacteria in the human mouth. It is among the first microorganisms to establish colonies within biofilms on mucous membranes and tooth surfaces, protecting epithelial cells from the adhesion and possible cytotoxic effects of pathogens [39, 40]. However, *S. oralis* acts as an opportunistic pathogen, causing endocarditis, meningitis, and eye infections. In addition, it can serve as a source of virulence genes for pathogens such as *S. pneumoniae* [36, 41]. There is no reliable evidence in the literature that this agent is one of the causative agents of tonsillopharyngitis. Importantly, accurate microbiological diagnosis within the *S. mitis* group is challenging because they are more than 99% identical in 16S rRNA sequence [42].

## 4. Conclusion

Multifactorial assessment of weather a species is a part of normal microbiota or a causative agent is needed in accordance to clinical symptoms, paraclinical tests, and immune competence. For precise microbiological diagnosis of opportunists, additional tests, such as agglutinations, automated biochemical systems, and molecular genetic methods, are needed. *S. anginosus* group, especially when exhibiting β-hemolysis, has to be considered as a causative agent of upper respiratory tract infections in adults.

## Data Availability

All datasets generated or analyzed during the study are included in the manuscript.
